# The rice TRIANGULAR HULL1 protein acts as a transcriptional repressor in regulating lateral development of spikelet

**DOI:** 10.1038/s41598-017-14146-w

**Published:** 2017-10-20

**Authors:** Peng Peng, Lihua Liu, Jingjing Fang, Jinfeng Zhao, Shoujiang Yuan, Xueyong Li

**Affiliations:** 1 National Key Facility for Crop Gene Resources and Genetic Improvement, Institute of Crop Science, Chinese Academy of Agricultural Sciences, Beijing, 100081 China; 2 Shandong Rice Research Institute, Jinan, 250100 China

## Abstract

As a basic unit of rice inflorescence, spikelet has profound influence on grain size, weight and yield. The molecular mechanism underlying spikelet development has not been fully elucidated. Here, we identified four allelic rice mutants, *s2-89*, *xd151*, *xd281* and *xd425*, which exhibited reduced width of spikelet, especially in the apical region. Map-based cloning revealed that all these mutants had missense mutation in the *TRIANGULAR HULL1* (*TH1*) gene, encoding an ALOG family protein. TH1 has been shown to regulate the lateral development of spikelet, but its mode of action remains unclear. Microscopic analysis revealed that the reduction in spikelet width was caused by decreased cell size rather than cell division. The TH1 protein was shown to localize in the nucleus and possess transcriptional repression activity. TH1 could form a homodimer and point mutation in the *s2-89*, *xd281* and *xd425* mutant inhibited homodimerization. The transcriptional repression activity of TH1 was partially relieved by the His129Tyr substitution in the *s2-89* mutant. Fusion of an exogenous EAR transcription suppression domain to the mutant protein TH1^s2-89^ could largely complemented the narrow spikelet phenotype. These results indicate that TH1 functions as a transcription repressor and regulates cell expansion during the lateral development of spikelet.

## Introduction

Rice (*Oryza sativa* L.), a model monocot with the smallest genome of major cereals, provides the staple food for over half of the world’s population. Increasing grain yield is a major target in the current rice breeding programs. Grain yield potential mainly depends on four major components: grain weight, panicle (or tiller) number per plant, grain number per panicle, and ratio of filled grains^1^. Grain weight is considerably determined by grain shape and size, which is specified by grain length, width, thickness and the length/width ratio.

Rice grain weight is nearly completely governed by genetic factors. With the advancement of the rice genome sequencing project and development of advanced mapping population, many quantitative trait loci (QTLs) for grain shape and size have been cloned using map-based cloning approach. Most of the cloned QTL genes affect grain size by influencing cell division in the spikelet hull. *GRAIN SIZE 3* (*GS3*), a major QTL for grain length and weight in rice, encodes a putative transmembrane protein, which functions as a negative regulator of grain and organ size^2,3^. *GRAIN LENGTH 3* (*GL3*), a novel serine/threonine phosphatase, regulates grain length by mediating cell division through affecting the phosphorylation status of cell cycle proteins^4^. *GRAIN WIDTH 2* (*GW2*), a QTL for rice grain width and weight, encodes a RING-type E3 ubiquitin ligase that negatively regulates cell division by targeting its substrates for degradation^5^. *GRAIN WIDTH* 5 (*GW5*), a major QTL underlying rice width and weight, encodes a novel nuclear protein which likely acts in the ubiquitin-proteasome pathway to regulate cell division during seed development^6^. *GRAIN WIDTH 8* (*GW8*) is synonymous with *OsSPL16*, which encodes a protein that is a positive regulator of cell proliferation. Higher expression of this gene promotes cell division and grain filling^7^. *GRAIN SIZE 5* (*GS5*) encodes a putative serine carboxypeptidase and functions as a positive regulator of grain width, such that higher expression of *GS5* is correlated with larger grain size^8^. In addition to cell division, cell expansion also plays important role in grain size. *POSITIVE REGULATOR OF GRAIN LENGTH 1* (*PGL1*) and *POSITIVE REGULATOR OF GRAIN LENGTH 2* (*PGL2*) positively regulate the rice grain length through increasing cell length in lemma and palea^9,10^. The studies above suggest that grain size is rigidly controlled by the size of spikelet hull.

Spikelet is a basic unit of the grass inflorescence architecture, whose development and morphogenesis has profound influence on grain size and weight. Grass species, such as wheat, rice, maize and barley, have a highly specialized inflorescence architecture which is apparently distinct from eudicots^11,12^. In rice, a spikelet is composed of two rudimentary glumes, two empty glumes (or sterile lemmas) and a floret which consists of one lemma, one palea, two lodicules, six stamens and one carpel^13–16^. Among these floral organs, lemma and palea play a critical role in determining the grain shape, size and yield, because the space enclosed by the lemma and palea determines the size of spikelet hull. Although the grass reproductive organs (stamens and carpels) appear to be largely conserved with eudicots, the non-reproductive floral organs (lemma, palea and lodicules) are considerably different from the sepals and petals found in flowers of most eudicots and many other monocots^17^. Currently, the widely-accepted ABCDE model describes the development of floral organs, which is based on early mutant studies in two eudicot species, *Antirrhinum majus* ^18^ and *Arabidopsis thaliana* ^19^. Some studies provide evidence in support of the hypothesis that grass lemma and palea are evolutionarily equivalent to eudicot sepals, whereas other studies suggest that lemma and palea should be regarded as bract and prophyll, respectively^20^. Hence, it is necessary to isolate the corresponding genes related to the origin and mechanism of lemma and palea development.

Genetic and molecular studies have allowed the identification of several mutants and genes that play key roles in regulating spikelet development through forward and reverse genetic approaches^12,13,15,21,22^. Spikelet development is controlled by the action of numerous transcription factors, the majority of which belong to the MADS-box family^14,23,24^. The AP1/FUL-like subfamily of MADS-box genes *RAP1B*/*OsMADS14* and *RAP1A*/*OsMADS15* have class A-like floral homeotic gene functions in specifying palea/lemma and lodicule identities^24^. The SEPALLATA(SEP)-like MADS gene *LEAFY HULL STERILE1* (*LHS1*)/*OsMADS1* specifies the identity of both the lemma and palea and regulates the determinacy of the spikelet meristem^22^. Another SEPALLATA (SEP)-like MADS gene, *PANICLE PHYTOMER2* (*PAP2*)/*OsMADS34* may act together with *ELE*/*G1* in controlling sterile lemma development^25–27^. The AGL6-like MADS-box gene *MOSAIC FLORAL ORGANS1* (*MFO1*)/*OsMADS6* gene, a major determinant of palea architecture, has a central role in spikelet development and is involved in floral meristem determinacy^28^. In addition, several grass-specific genes also play key roles in regulating spikelet development. The *DEPRESSED PALEA1* (*DP1*) gene encodes a nuclear-localized AT-hook DNA binding protein and plays a grass-specific role of chromatin architecture modification in flower development^29^. The *RETARDED PALEA1* (*REP1*) gene has a specific role in the regulation of palea development. The reduced cell size in the palea of the *rep1* mutant implies a role of *REP1* similar to other TCP proteins in regulating cell expansion and differentiation^30^. Further study found that *REP1* is downstream of and regulated by *DP1* ^29^. In addition, the rice *EXTRA GLUME1* (*EG1*) gene, a putative lipase gene, specifies empty glume fate and floral meristem determinacy^31^. The LONG STERILE LEMMA1 (G1) protein contains an ALOG domain and belongs to a recently described class of transcription factor. The *g1* mutant shows the striking phenotype of sterile lemmas transformed into lemmas^32^. Thus, mutations in the genes mentioned above caused dramatic abnormality in the spikelet. By contrast, in the *triangular hull1* (*th1*) mutant, the floral organ identity and patterning are normal, but the lemma and palea become narrower especially in the apical region, forming a beak-like spikelet^33,34^. Several allelic mutants of *th1* named *beak like spikelet1* (*bls1*), *beak-shaped grain1* (*bsg1*) and *abnormal flower and dwarf1* (*afd1*) were also reported^35–37^. The *TH1* gene encodes a nuclear protein with a conserved ALOG domain of unknown function^32,33^. It is still unclear how *TH1* specifically regulates the lateral development of the lemma and palea.

In the present study, we isolated four allelic mutants of *TH1*, namely, *s2-89*, *xd151*, *xd*281 and *xd425* from the EMS-mutagenized *japonica cv*. Nipponbare and Xu Dao3, respectively. Detailed morphological analysis indicates that the reduction in spikelet width was caused by decreased cell size rather than cell division in the lemma and palea. Cellular and biochemical studies show that the TH1 protein is localized in the nucleus, functions as a homodimer, and has transcription repression activity. Point mutations in the ALOG domain disrupted dimerization and compromised its transcription repression activity. Phenotypic analysis of transgenic plants harboring the 4 × EAR-TH1^s2-89^ or 4 × VP16-TH1^s2-89^ fusion constructs further supported that TH1 functions as a transcriptional repressor. This work provides a new perspective of TH1 function in regulating spikelet development and biochemical function of the ALOG family proteins.

## Results

We identified four allelic mutants of *TH1*, i.e., *s2-89*, *xd151*, *xd281* and *xd428*. Because they show similar phenotypes, we mainly describe *s2-89* below.

### Morphological phenotype of the *s2-89* mutant

**Figure 1:**
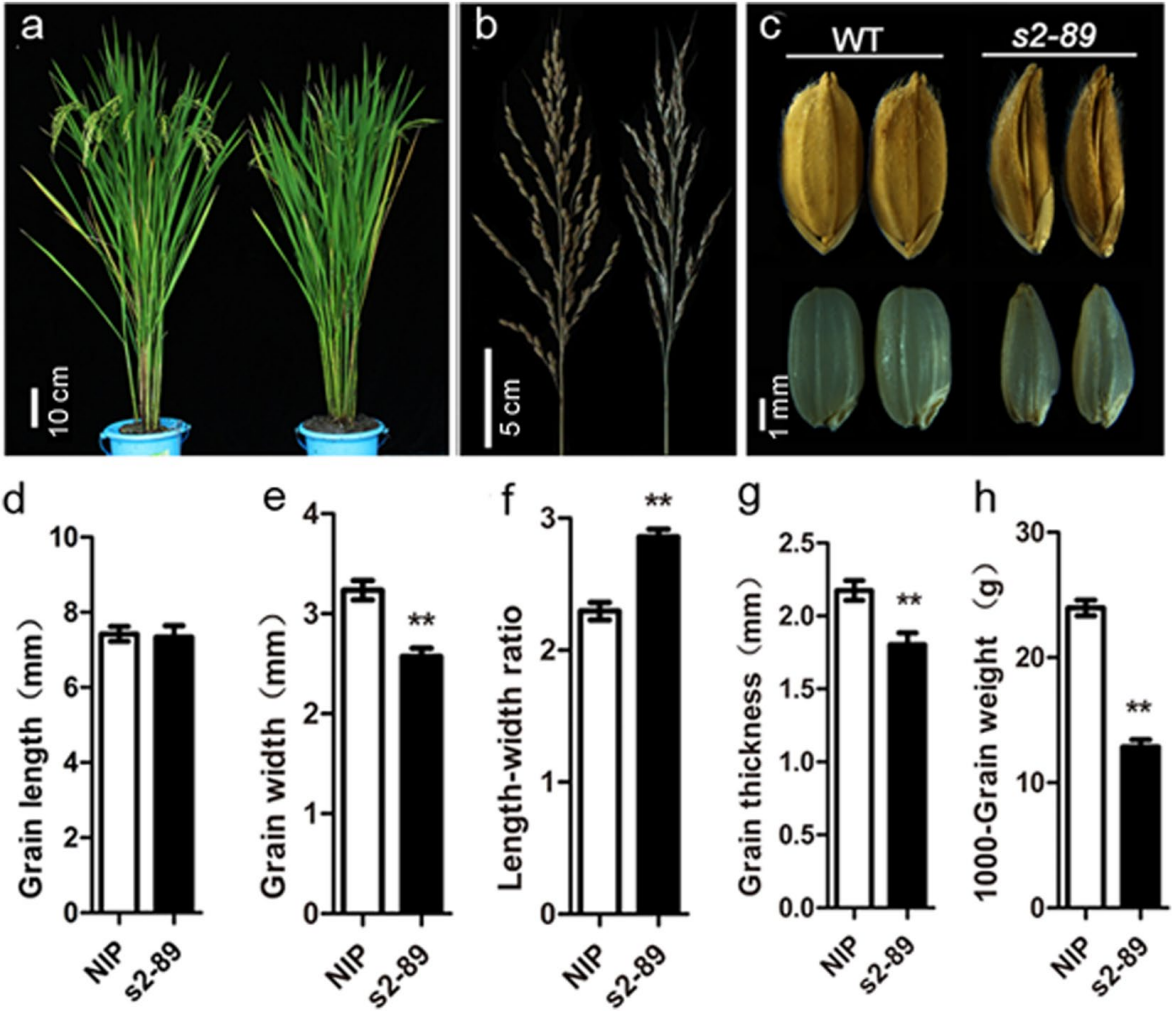
Morphological phenotypes of the *s2-89* mutant (right) and its wild-type Nipponbare (left). (**a**) Whole plant phenotype at grain-filling stage. (**b**) Panicle morphology. (**c**) Grain morphology. The upper row: unhulled seeds; the lower row: hulled seeds. (**d**–**h**) Quantification of grain length, width, length-width ratio, thickness, and 1000-grain weight, Data are given as mean ± SE (n = 10). Double asterisks indicate significant differences between WT and *s2-89* at P < 0.01 by Student’s t test. Each scale bar is indicated.

The *s2-89* mutant was isolated from the M_2_ population of *japonica cv*. Nipponbare mutagenized by the ethyl methane sulphonate (EMS). Compared with the wild type, the *s2-89* mutant showed no apparent difference in the vegetative phase. At maturity, phenotype of the *s2-89* mutant was also not conspicuously different from that of the wild type in many respects such as plant type, panicle structure and other agronomic traits (Fig. 1a,b, see Supplementary Table S1). The most striking phenotype of the *s2-89* mutant was the abnormal grain morphology. The unhulled grain of the *s2-89* mutant exhibited a pointed beak-like shape and the hulled grain displayed a triangle-like shape (Fig. 1c). After measuring the grain traits, we found that the *s2-89* mutant is reduced in grain width and thickness relative to the wild type, whereas there is no significant change in grain length, which leads to the increased grain length-width ratio. In addition, the 1000-grain weight of the *s2-89* mutant is only 53.72% of that of wild type (Fig. 1d–h, see Supplementary Table S1). These results collectively indicated that grain weight is affected predominantly by the abnormal grain morphology.

In addition to the *s2-89* mutant, we obtained other three similar beak-shaped grain mutants from EMS-mutagenized japonica cultivar Xu Dao3, referred as *xd151*, *xd281* and *xd425* (see Supplementary Figure S2a). The grain width of these mutants was reduced by 4.36%, 8.72% and 9.66% of that of wild type, respectively. The grain thickness of these mutants was reduced to varying degrees by 4.44%, 6.22% and 11.56%, respectively. However, the grain length of these mutants was increased by 11.75%, 11.05% and 0.84% of that of wild type, respectively. Consistent with the grain size, the 1000-grain weight of these mutants was also reduced (see Supplementary Figure S2b–f, Table S2).

### Comparison of cell size and cell number in WT and *s2-89* spikelet hull

**Figure 2:**
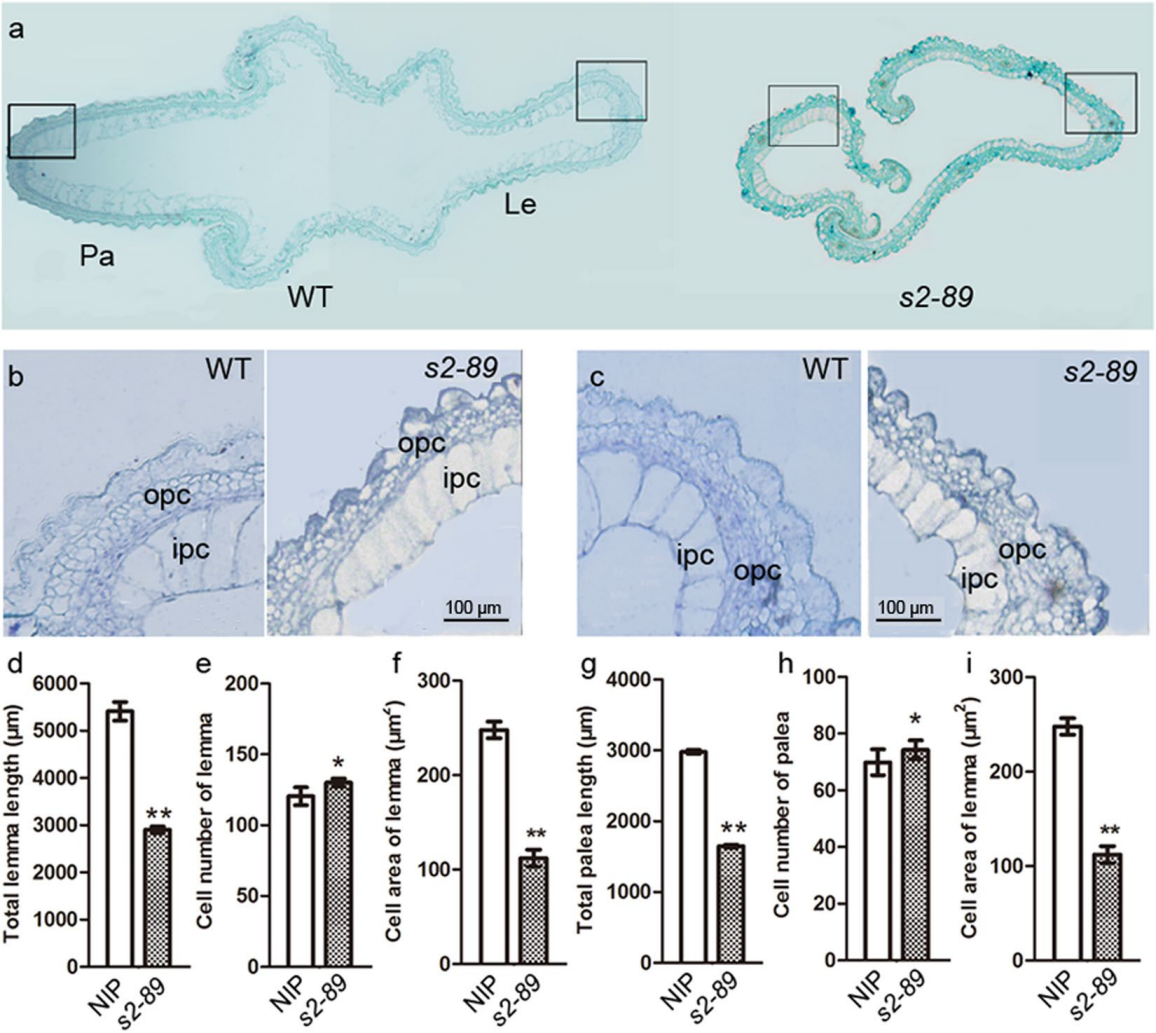
Histological analyses of the WT and *s2-89* mutant spikelet at booting stage. (**a**) Transverse sections at the apical region of the spikelet hulls. Pa: palea; Le: lemma. (**b**) Close-up views of the cross-section boxed in (**a**) of palea. ipc: inner parenchymal cell; opc: out parenchymal cell. (**c**) Close-up views of the cross-section boxed in (**a**) of lemma. **(d**–**f**) Comparison of total length, cell number and cell area in the inner parenchymal cell layers of palea of WT and *s2-89*. (**g**–**i**) Comparison of total length, cell number and cell area in the inner parenchymal cell layers of lemma of WT and *s2-89*. Data are given as mean ± SE (n = 10). Double asterisks and asterisk indicate significant differences between WT and *s2-89* at P < 0.01 and P < 0.05 by Student’s t test. Each scale bar is indicated.

Given that the slender grain phenotype of the *s2-89* mutant was more obvious in the apical than the middle region, we compared the cross-sections of the *s2-89* and wild type spikelet hull at the apical region (Fig. 2a–c). The perimeter length of both lemma and palea was significantly reduced at the apical region (Fig. 2d and g). Both palea and lemma comprise outer parenchyma cell layer (opc) and inner parenchyma cell (ipc) layer (Fig. 2b,c). Because the inner parenchyma cells are regular in shape and large in size, we counted the number of inner parenchyma cells and measured their size. Although the grain width is reduced, the number of inner parenchyma cells along the grain width direction was slightly increased in both the lemma and palea of the *s2-89* mutant (Fig. 2e and h). However, size of the inner parenchyma cell was reduced by about 50% (Fig. 2f and i). These observations demonstrated that the reduced width of the *s2-89* spikelet hull was mainly due to the decrease in cell size.

**Figure 3:**
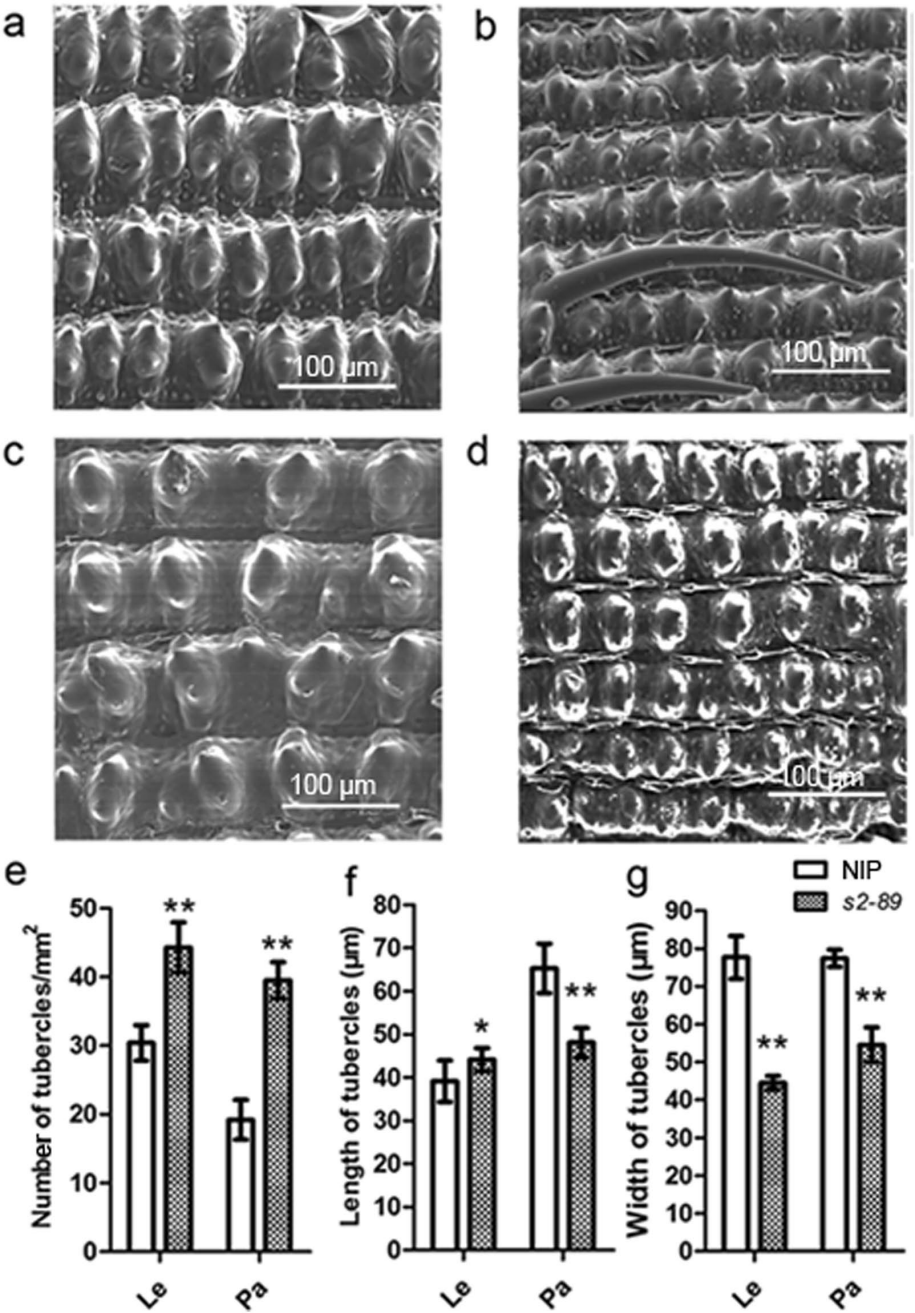
SEM observation of the outer surface of the wild type and *s2-89* spikelet. (**a**,**b**) SEM analysis of the outer surface of the WT (**a**) and *s2-89* (**b**) lemmas. (**c**,**d**) SEM analysis of the outer surface of the WT (**c**) and *s2-89* (**d**) paleas. (**e**) The average number of tubercles per mm^2^ on the outer surface of palea (Pa) and lemma (Le). Double asterisks indicate significant differences between WT and *s2-89* at P < 0.01 by Student’s t test. (**f**,**g**) The average length and width of tubercles. Data are given as mean ± SE. (*n* = 10). Double asterisks and asterisk indicate significant differences between WT and *s2-89* at P < 0.01 and P < 0.05 by Student’s t test. Each scale bar is indicated.

To further reveal the characteristics of the spikelet in the *s2-89* mutant, the lemma and palea of the wild type and *s2-89* mutant spikelets were observed through scanning electron microscopy (SEM). These two organs have a rough outer epidermal surface covered with convex structures named tubercles^32^. The average number of tubercles per unit area of outer epidermal surface of lemma and palea in the *s2-89* mutant was increased substantially than the wild type (Fig. 3a–e). The average length and width of tubercle in the *s2-89* mutant decreased significantly, compared with that of wild type (Fig. 3f,g). These results indicate that the decrease in cell size was mainly responsible for the pointed beak-shaped grain of this mutant.

Both cell proliferation and cell expansion is essential for the control of organ size^38^. Cyclin B 1;1 (CYCB1; 1) is strongly expressed in cells with high mitotic activity^39^, and has been used as a marker of G_2_/M of the cell cycle. To examine whether cell division activity is reduced in the spikelet hull of the *s2-89* mutant, we compared the CYCB1;1-GUS staining pattern in the wild type and *s2-89* mutant background, respectively. There was no obvious difference in the GUS staining pattern between the mutant *s2-89* and wild type (see Supplementary Figure S3). This result suggests that cell division may not be responsible for the narrower spikelet hull in the *s2-89* mutant.

### The *s2-89* mutant is a novel allelic mutant of *TH1*

**Figure 4:**
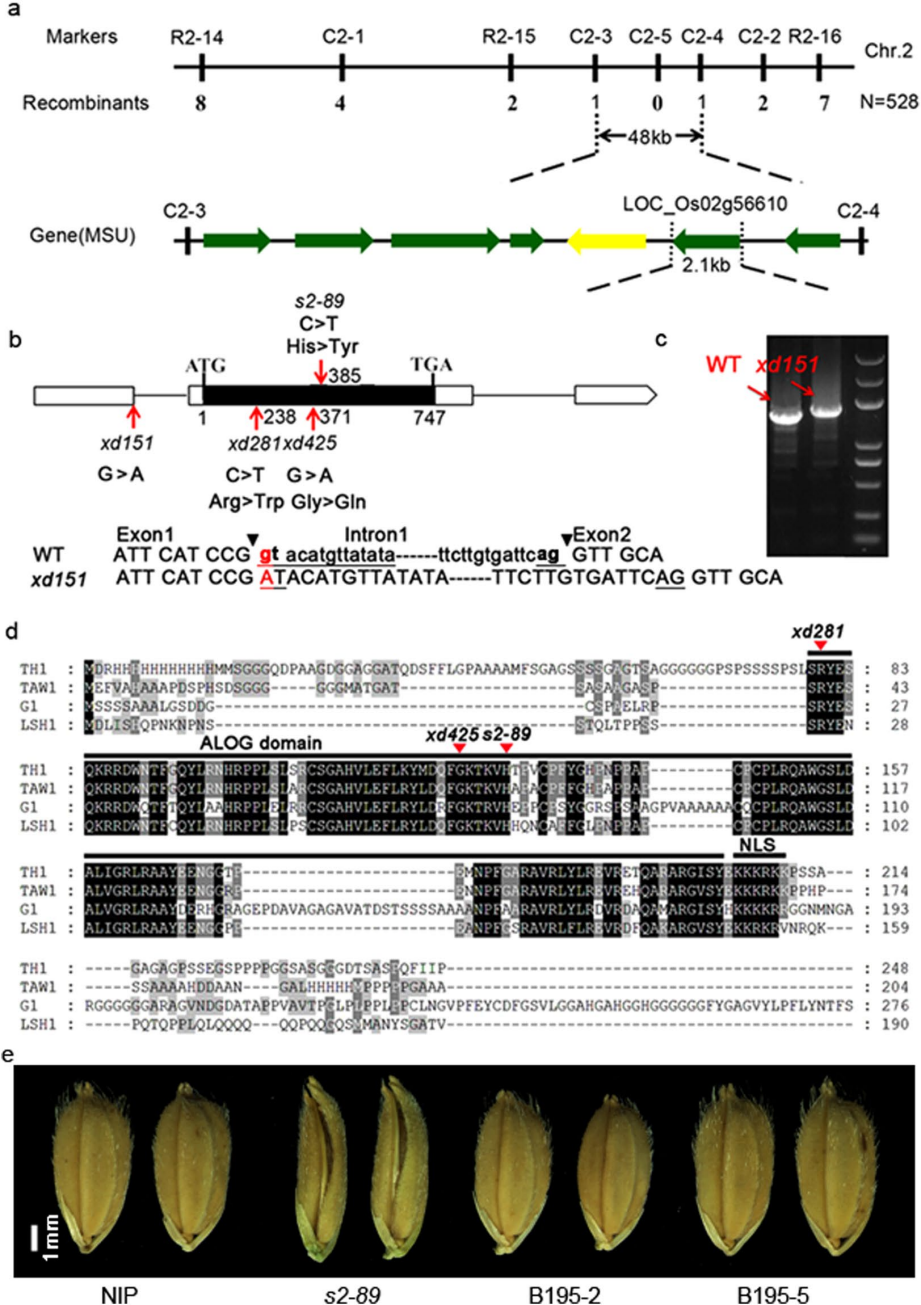
Identification and characterization of the *TH1* gene. (**a**) The *TH1* locus was mapped to a region between markers R2-15 and R2-16 on chromosome 2. The gene was further delimited to a 48-kb genomic region between markers C2-3 and C2-4. There are seven predicated ORFs within this region. (**b**) The *TH1* gene structure. White and black box represent untranslated region and coding sequence, respectively. Lines represent intron. The start codon (ATG) and the stop codon (TGA) are indicated. The *xd151*, *xd281*, *xd425* and *s2-89* mutation sites in the *TH1* gene are shown. (**c**) Comparison of the size of *TH1* cDNA between WT and the *xd151* mutant. The first intron was retained within the mRNA transcript. (**d**) Sequence alignment of the TH1 protein with three functionally characterized member of the ALOG family, i.e., Arabidopsis LSH1, rice TAW1 and G1. The conserved ALOG domain and nuclear localization sequence (NLS) are underlined. Mutations in × *281*, *xd425* and *s2-89* are indicated by red arrowhead. (**e**) Complementation of grain morphology. Two transgenic lines B195-2 and B195-5 are shown in which the wild type *TH1* genomic DNA was introduced into the *s2-89* mutant.

To investigate the inheritance pattern, the *s2-89* mutant was crossed with the wild type Nipponbare. In the F_2_ population, the ratio of plants with normal grain to those with beak-like shape grain was 208:65, which was fit for the ratio of 3:1 (χ^2^ = 0.148 < χ^2^_0.05_ = 3.84), indicating that the mutant phenotype was controlled by a single recessive gene. To identify the gene, we used a map-based cloning approach. Primary mapping using 32 F_2_ mutant individuals derived from a cross between the *s2-89* mutant and the *indica* variety Dular revealed that the mutation locus localizes between two insertion-deletion (InDel) markers R2-14 and R2-16 on chromosome 2. For fine mapping, we developed six new InDel markers and the gene was narrowed to a 48 kb interval between molecular markers C2-3 and C2-4 (Fig. 4a).

Based on the available MSU Rice Genome Annotation Project (http://rice.plantbiology.msu.edu/), seven predicted candidate genes were located in this region. Among them, the *LOC_Os02g56610* locus has been proved to be the *TH1*/*BSG1*/*BLS1*/*AFD1* gene in several previous studies^33,35–37^. The *th1*/*bsg1*/*bls1*/*afd1* mutants produce narrow spikelet, similar to the *s2-89* mutant. To test whether the *s2-89* mutant had a mutation in the *TH1* gene, the candidate gene was amplified and sequenced from both *the s2-89* mutant and wild type genomic DNA. A transition from C to T in the coding sequence of the *TH1* gene was detected, resulting in a change of the 129th amino acid from His to Tyr (Fig. 4b). We further sequenced the *TH1* gene from the other three mutants *xd151*, *xd281*, *xd425* and the corresponding wild type Xu Dao3. In the *xd151* mutant, a transition from G to A occurred at the 5′ splicing site of the first intron. RT-PCR amplification of cDNA revealed that the *TH1* transcript became larger in the *xd151* mutant than wild type (Fig. 4c). Sequence analysis confirmed that the first intron was retained within the 5′-UTR of the *TH1* gene (Fig. 4b, lower panel). In the *xd281* mutant, a transition from C to T was detected, causing the 80th amino acid Arg to be substituted by Trp. In the *xd425* mutant, a transition from G to A was found, resulting in a change of the 124th amino acid from Gly to Gln (Fig. 4b). Furthermore, in a functional complementation experiment, transformation of the wild-type *TH1* genomic fragment into the *s2-89* mutant was able to rescue the beak-shaped grain phenotype (Fig. 4e). Based on these results, we concluded that mutations in *TH1* are responsible for the beak-shaped grain phenotype.

The *TH1* gene encodes a protein with a conserved ALOG domain of unknown function. Sequence alignment of TH1 with three previously identified members of the ALOG family, i.e., *Arabidopsis* LIGHT-DEPENDENT SHORT HYPOCOTYLS1 (LSH1), rice LONG STERILE LEMMA (G1) and TAWAWA1 (TAW1)^32,40,41^, showed that the central ALOG domain is highly conserved whereas the N- and C-terminus are highly diverse (Fig. 4d). The three missense mutations in the *s2-89*, *xd281* and *xd425* mutant are present in the ALOG domain (Fig. 4d). In the quantification analysis of grain related traits, grains of these three allelic mutants were found to be more severely distorted than that of the *xd151* mutant (see Supplementary Figure S2), which has an intact ALOG domain (Fig. 4b). This result suggests that these amino acids and the ALOG domain are critical for the proper function of the TH1 protein.

### Expression patterns of *TH1*

We used real-time PCR analysis to examine the spatial expression pattern of *TH1*. The result indicated *TH1* was highly expressed in young panicles, especially in the lemma and palea of spikelets. Although *TH1* was also expressed in other tissues such as root, culm, leaf blade, leaf sheath and tiller bud, the expression level was relatively lower (see Supplementary Figure S4). This result indicated that *TH1* is predominantly expressed in a spikelet-specific manner, which is consistent with the fact that the *s2-89* mutant exhibited abnormal phenotype mainly in the spikelet. This result is also consistent with a previous *TH1* promoter driven GUS reporter assay where strong GUS signals were detected in the lemmas and paleas of young spikelets^31^. In another assay using *in situ* hybridization, *TH1* was shown to be highly expressed in the primordia of the lemma and palea and also detected in the primordia of the rudimentary glume, sterile lemma, lodicule and stamen^29^.

### Nuclear localization and dimerization of TH1

**Figure 5:**
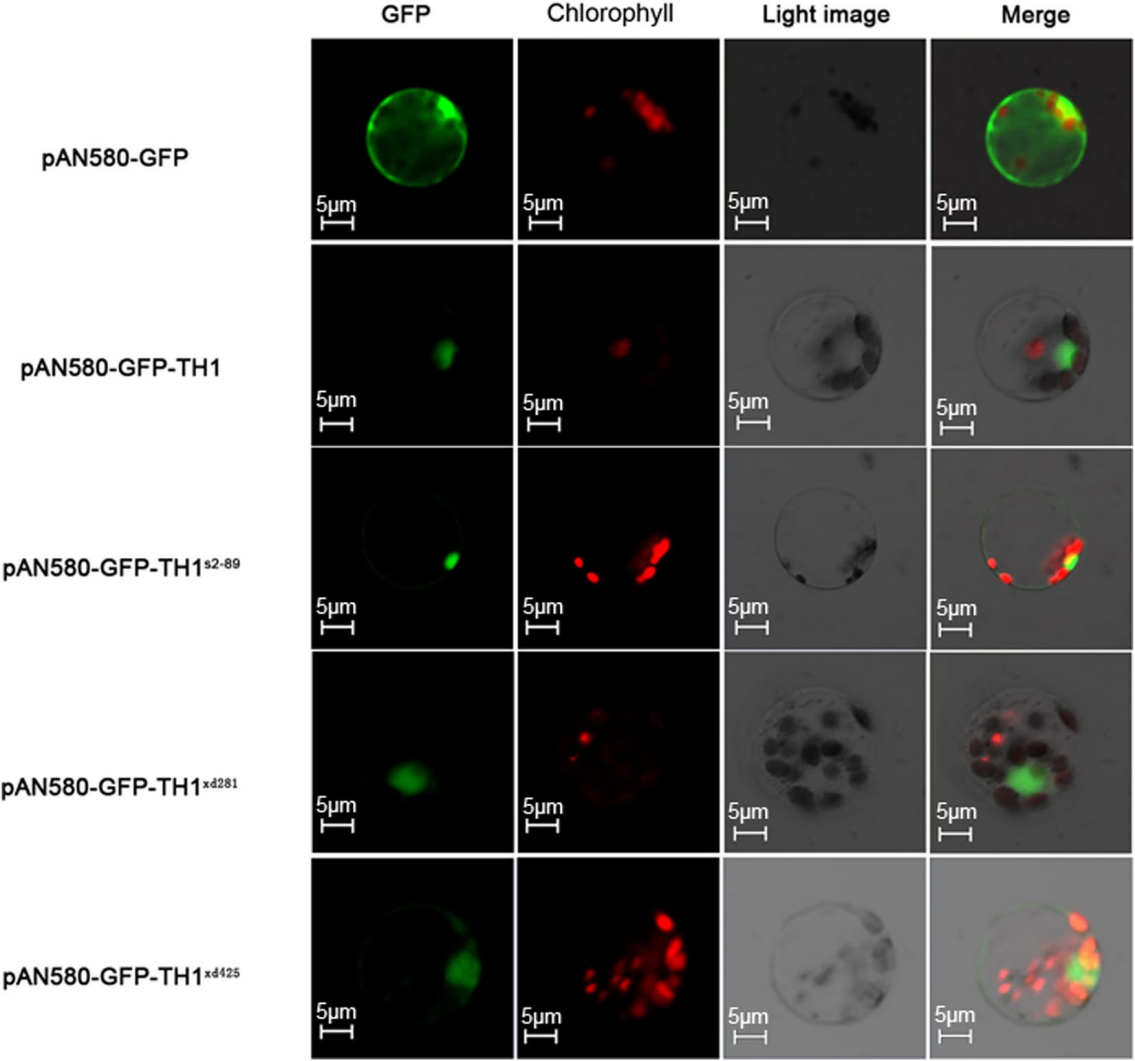
Subcellular localization of the wild type and mutant TH1 protein. The pAN580-GFP-TH1, pAN580-GFP-TH1^s2-89^, pAN580-GFP-TH1^xd281^, pAN580-GFP-TH1^xd425^ vector was transformed into protoplasts prepared from rice seedlings. The pAN580-GFP vector was used as control. Each scale bar is indicated.

Previous bioinformatic study has predicted that the ALOG domain proteins may act as transcription factors or recruiters of repressive chromatin^42^. If TH1 acts as a transcription factor, nuclear localization is a prerequisite to execute its function. A nuclear localization signal (KKKRKK) was predicted immediately after the ALOG domain (Fig. 4d), using the web-based tools such as NucPred^43^ and WolFPSORT^44^. To test this possibility, the wild type and mutant alleles of the *TH1* gene were fused in-frame with the green fluorescent protein (GFP) under the control of the CaMV35S promoter in the transient expression vector pAN580. The fusion vectors were transformed into rice leaf protoplasts. The fluorescent signal of TH1-GFP was targeted mainly to the nucleus. Interestingly, green fluorescence of the three mutant TH1 proteins, i.e., TH1^s2-89^-GFP, TH1^xd281^-GFP and TH1^xd425^-GFP was also mainly detected in the nucleus (Fig. 5). This result indicates that these three substitutions have no effect on the TH1 subcellular localization.

**Figure 6:**
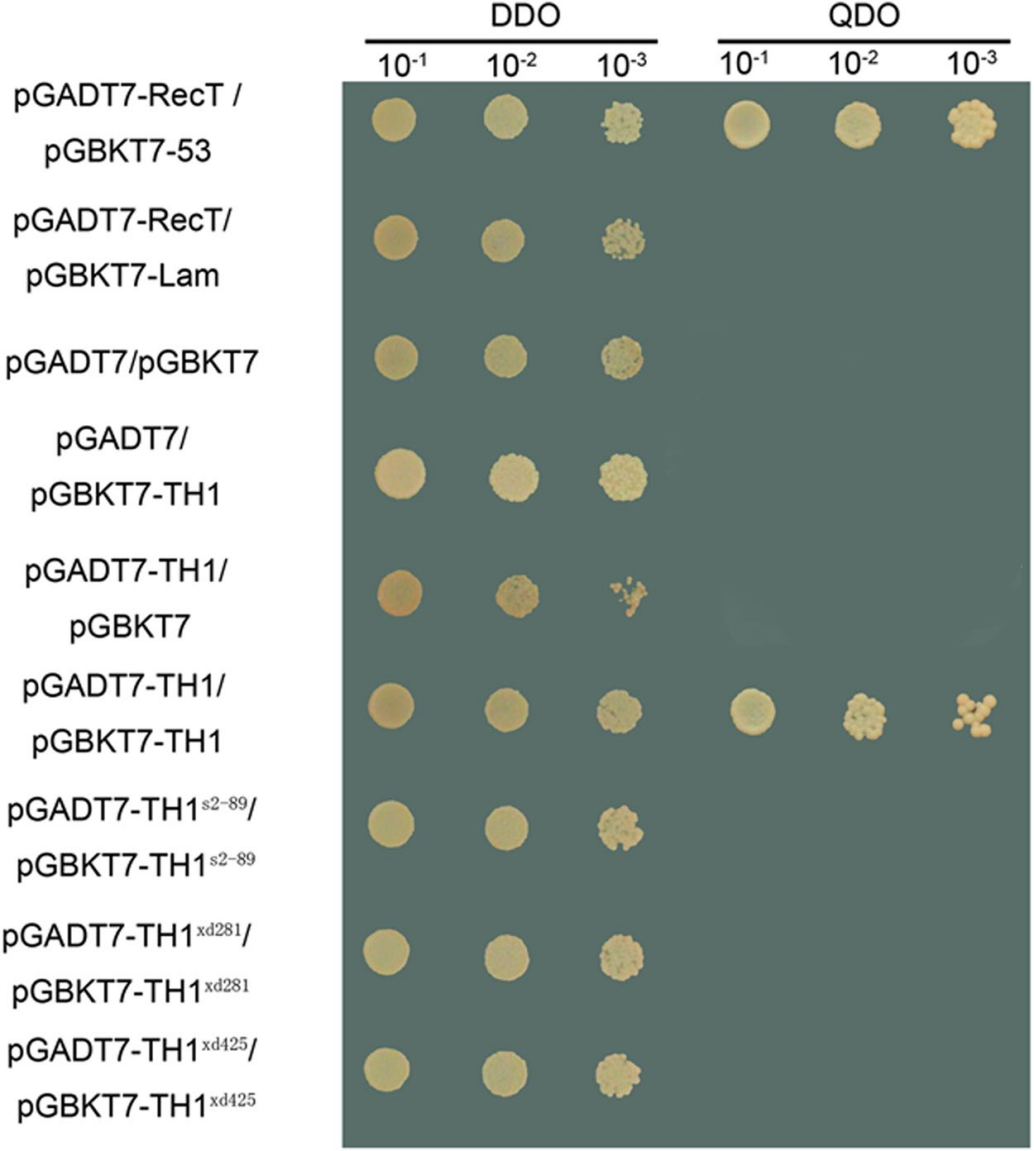
Homodimer formation of TH1 protein detected via yeast two-hybrid assay. The WT and three mutant alleles of the TH1 gene were cloned into both the bait vector pGBKT7 and the prey vector pGADT7 and co-transformed into the yeast strain Y187. A decimal serial dilution of the yeast colony was spotted onto the selective medium DDO (SD/-Trp-Leu) or QDO (SD/-Trp-Leu-Ade-His). The colony will survive on the QDO medium only if the bait and prey interact with each other. pGADT7-RecT and pGBKT7-53 was used as positive control, while pGADT7-RecT and pGBKT7-Lam as negative control.

Considering the fact that transcription factors usually function as a homodimer^45^, we examined whether TH1 can form a homodimer using the yeast two-hybrid assay. As shown in Fig. 6, interaction between BD-TH1 and AD-TH1 was detected, suggesting that TH1 can interact with itself to form a homodimer. However, the three TH1 mutant proteins TH1^s2-89^ (His129Tyr), TH1^xd281^ (Arg80Trp) and TH1^xd425^ (Gly124Gln) lost the ability to interact with itself. Therefore, Arg80, Gly124 and His129 are critical for the homodimerization of TH1.

### TH1 acts as a transcriptional repressor in regulating spikelet development

**Figure 7:**
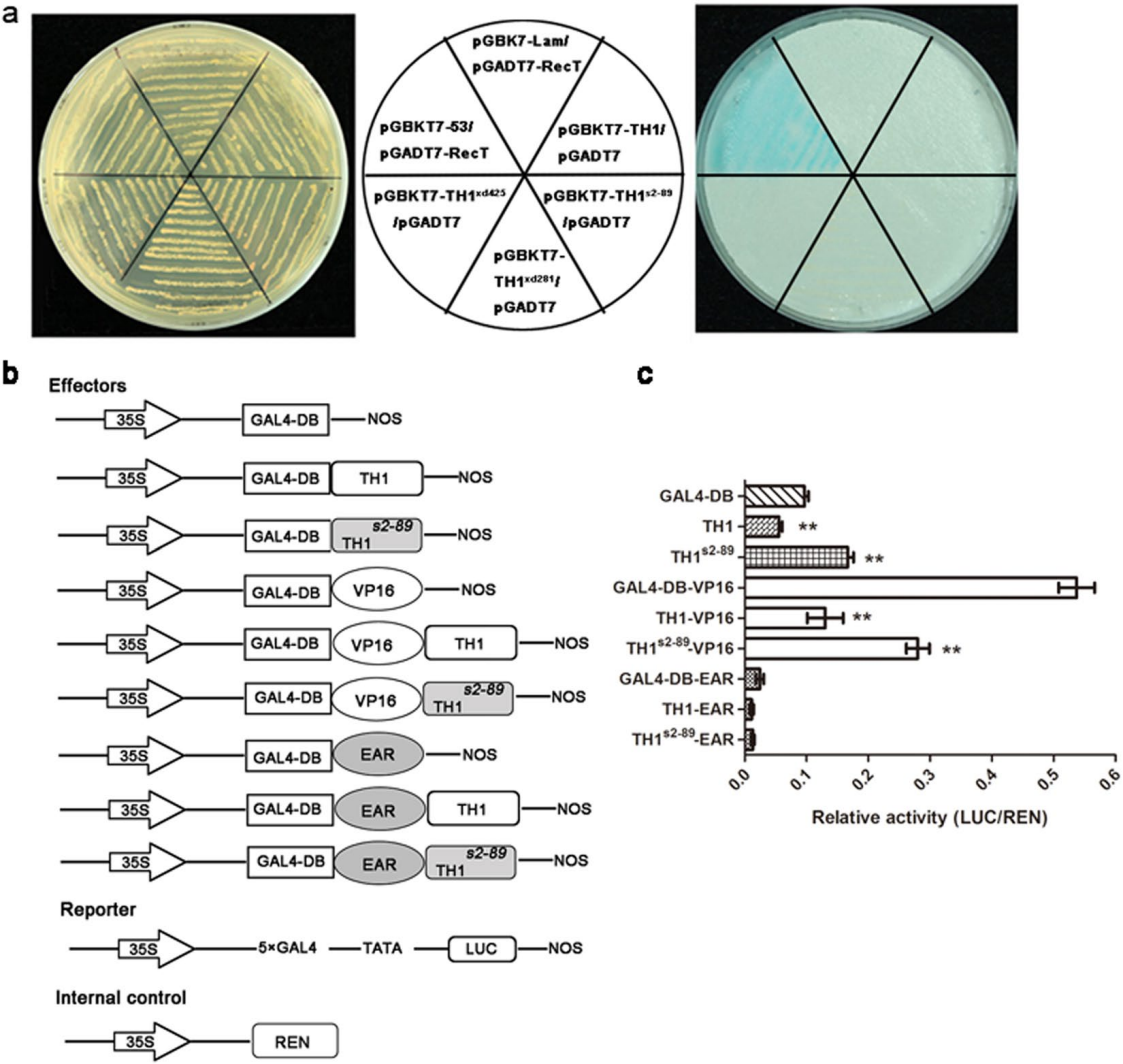
TH1 acts as a transcriptional repressor in the transient expression assay. (**a**) Transcriptional activation assay in yeast cell. Yeast co-transformants were incubated on the selective medium (SD/-Ade/-His/-Leu/-Trp plus X-α-Gal) at 30 °C for 3 d. If TH1 is a transcription activator, the β-Galactosidase reporter gene will be expressed and the filter will be stained blue in the Colony-lift Filter Assay. pGADT7-RecT & pGBKT7-53 (positive control); pGADT7-RecT & pGBKT7-Lam (negative control). (**b**) Constructs used in transient expression assay in rice leaf protoplasts. Six different effector plasmids were made by fusing TH1, TH1^S2-89^ with activator domain VP16, repressor domain EAR or the GAL4 DNA binding domain (GAL4-DB). The reporter plasmid Pro35S-GAL4:LUC contains a CaMV 35 S promoter, 5 × GAL4 binding site, a TATA box in front of the firefly *luciferase* (*LUC*) gene and a nopaline synthase terminator. The p35S: REN plasmid expressing the *Renilla* luciferase was used as an internal control. (**c**) Relative luciferase activities in rice leaf protoplasts using TH1, TH1^s2-89^, VP16, TH1-VP16, TH1^s2-89^-VP16, EAR, TH1-EAR and TH1^s2-89^-EAR as effector compared with GAL4-DB control. Error bars indicate SE (*n* = 3). Double asterisks indicate significant difference at P < 0.01 by Student’s t test.

To test whether TH1 possesses the capacity to activate transcription, a fusion of TH1 coding region with the yeast GAL4 DNA binding domain was expressed in the yeast strain Y187 containing the β-galactosidase reporter gene driven by the GAL4 UAS (GAL4 binding site). If TH1 is a transcription activator, the β-Galactosidase reporter gene will be expressed and the filter will be stained blue in the Colony-lift Filter Assay. However, we could not detect any β-Galactosidase activity using either the wild type or mutant TH1 protein, although it was easily detected in the positive control (Fig. 7a). These results suggest that TH1 could not act as a transcriptional activator, at least in yeast.

To determine whether TH1 acts as a transcriptional repressor, we employed the luciferase transient expression assays in rice protoplasts. For the effector plasmids, coding sequence of the wild type TH1 and mutant TH1^s2-89^ was inserted into the p35S-GAL4DB plasmid^46^ to generate fusion proteins with the GAL4 DNA binding domain (GAL4 DB). The reporter plasmid contains a CaMV 35 S promoter, 5 × GAL4 UAS (GAL4 binding site), a minimal TATA region and the firefly luciferase gene (Fig. 7b). Each effector plasmid was co-transformed with the reporter plasmid into rice protoplasts and luciferase activity was measured. As a control, fusion of the activation domain of the herpes simplex virus VP16 protein^47^ or the EAR transcription repression domain^43^ to GAL4DB resulted in enhanced or reduced luciferase activity, respectively, compared with GAL4 DB alone (Fig. 7c). Interestingly, fusion of TH1 with GAL4 DB or GAL4DB-VP16 resulted in about two-fold or three-fold reduction in luciferase activity, indicating that TH1 has transcription repressive activity (Fig. 7c). However, the substitution of His129Tyr in TH1^s2-89^ relieved the repression activity. Fusion of TH1^s2-89^ with GAL4 DB increased rather than decreased luciferase activity. Fusion of TH1^s2-89^ with GAL4DB-VP16 reduced luciferase activity to a less extent (Fig. 7c). The loss of transcription repressive activity of the TH1^s2-89^ mutant protein is probably due to the lack of homodimerization of TH1^s2-89^ (Fig. 6). However, we can not rule out the possibility that the s2-89 mutation may alter domain folding thus affect protein function.

**Figure 8:**
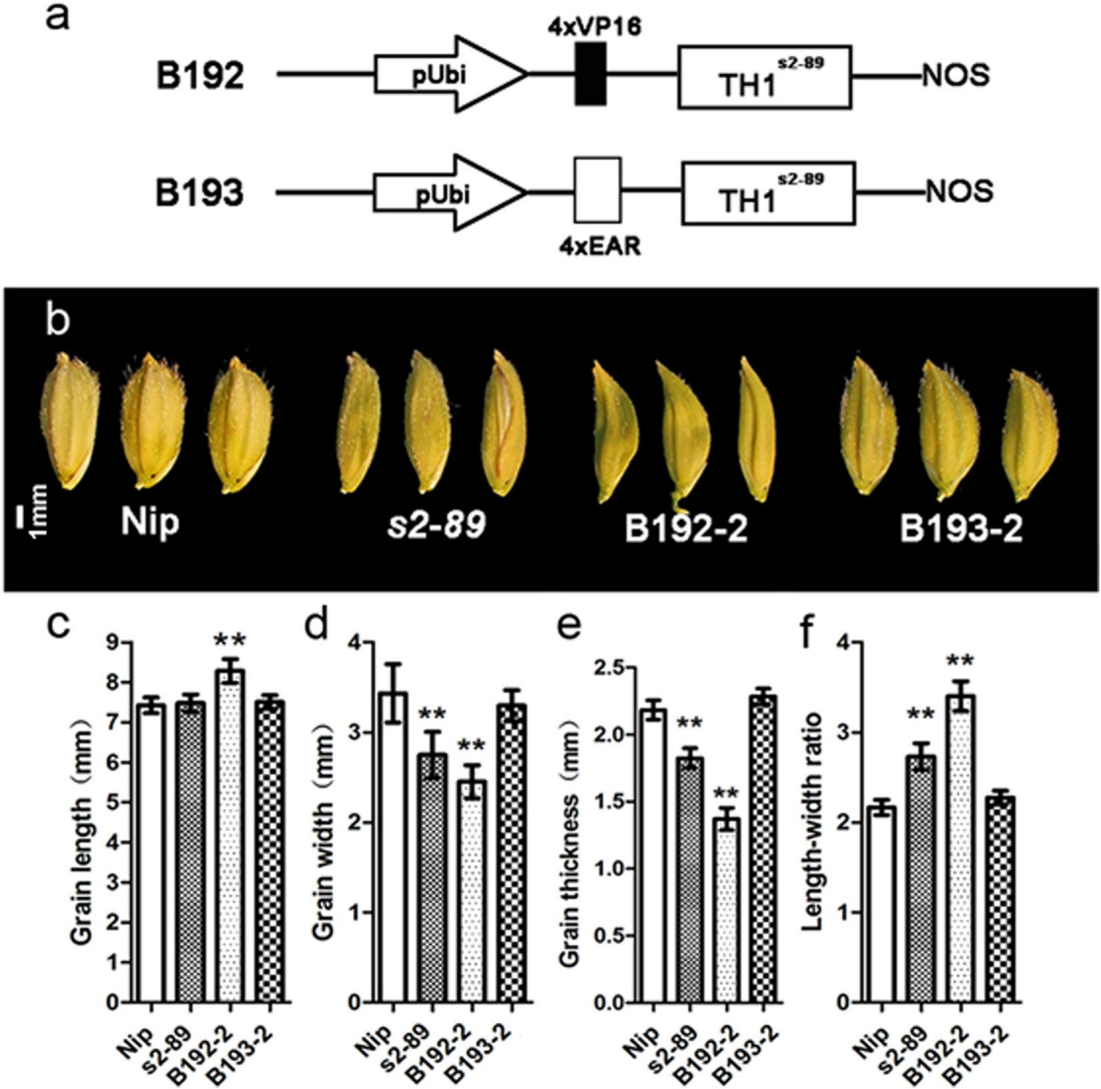
Requirement of the TH1 transcriptional repression activity in spikelet development. (**a**) Schematic representation of the vectors transformed into the *s2-89* mutant. Four copies of transcription activation domain VP16 or repression domain EAR were fused with the TH1^s2-89^ mutant protein. (**b**) Spikelet morphology of transgenic lines expressing the VP16-TH1^s2-89^ fusion protein (B192-2) and the EAR-TH1^s2-89^ fusion protein (B193-2) in *s2-89* mutant. (**c**) Comparisons of grain length, grain width, length-width ratio and grain thickness in WT, *s2-89* and the transgenic lines. Data are means ± SE (n = 20). Double asterisks indicate significant differences at P < 0.01 by Student’s t test. Each scale bar is indicated.

To confirm the requirement of the TH1 transcriptional repression activity in spikelet development, we performed a complementation experiment in the *s2-89* mutant. The exogenous EAR transcription suppression domain^48^ and VP16 transcription activation domain^47^ was fused to the N-terminus of the mutant protein TH1^s2-89^ (Fig. 8a), respectively. These constructs were transformed into the *s2-89* mutant. The *EAR-TH1* ^*s2-89*^ transgenic plants largely complemented the spikelet and grain phenotype of the *s2-89* mutant (Fig. 8b–f). Consistent with the transgenic phenotype, the TH1^s2-89^-EAR fusion protein exhibited transcription repressive activity in luciferase transient expression assays in rice protoplasts (Fig. 7c). Therefore, fusion of an exogenous transcription suppression domain to the mutant protein TH1^s2-89^ restores the complementation ability. In contrast, transgenic plants expressing *VP16*-*TH1* ^*s2-89*^ exhibited more slender spikelet phenotype, compared with the wild type and mutant control (Fig. 8b–f). These results indicate transcription repression activity of TH1 acts to promote the lateral development of rice spikelet.

## Discussion

Rice grain size is an important agronomic trait and nearly completely governed by genetic factors. Several genes involved in rice grain development have been isolated, such as *GS3*, *GL3*, *GW2*, *GW5* and *GW8* ^2–8^. These studies suggest that spikelet architecture has profound influence on grain size and weight. In this study, we isolated four grain shape related mutants, *s2-89*, *xd151*, *xd281* and *xd425*, which showed an unusually slender spikelet. Map-based cloning approach revealed that these mutants have single base mutation in the *TH1* gene, which is previously reported to function in the control of rice spikelet shape and grain size^33–37^. In this study, we made two novel insights about the function of *TH1*. Firstly, *TH1* determines grain shape and size by regulating cell expansion in the lemma and palea of rice spikelet. Secondly, TH1 functions as a transcriptional repressor in spikelet development.

Each rice spikelet consists of a floret with one carpel, six stamens and two lodicules subtended by the lemma and palea, and two depressed empty glumes at a position opposite to each other above the rudimentary glumes. It was previously reported that TH1 has a specific role in regulating lemma and palea development^33–37^. In our study, the *s2-89*, *xd151*, *xd281* and *xd425* mutants bore beak-shaped grains with reduced width, thickness and weight (Fig. 1, Supplementary Figure S2), as for other mutant alleles of *TH1* ^33–37^. Young florets in the *th1* mutant and corresponding wild type at different developmental stages had been observed by SEM. *TH1* seems not to influence the initiation of floral-organ primordia, but affects the enlargement of the lemma and palea at late development stage of spikelet^35,36^. Meanwhile, in the *th1* mutants, the marginal region of palea (mrp) looks normal and the alterations are due to shape changes of the lemma and the rest of palea (body of palea, bop)^36^. In addition, the lodicules of *th1* florets were elongated and green^34,36,37^. Further observations showed that the surface of elongated lodicules was rough, crumpled and possessed some protrusions that were similar with those in the wild type hulls^36^. In this study, we determined whether the reduced spikelet size is caused by regulations of cell size or cell number in *th1* spikelet. Our histological observation showed that the *s2-89* mutant exhibited smaller inner parenchyma cell in both the lemma and palea, compared with the wild-type (Fig. 2). The number of inner parenchyma cell was slightly increased rather than decreased in the *s2-89* mutant (Fig. 2). SEM observation also revealed that the average length and width of tubercle on the outer surface of lemmas and paleas was decreased significantly in the mutant *s2-89*, compared with that of wild-type (Fig. 3). Moreover, the similar staining pattern of the CYCB1;1-GUS mitotic marker in the wild type and *s2-89* mutant background indicated cell division may not be responsible for the narrower spikelet in the *s2-89* mutant (see Supplementary Figure S3). To sum up, the reduced width of the *th1* spikelet hull resulted mainly from the decrease in cell size. TH1 most likely functions as an upstream modulator of cell expansion during lateral development of lemma and palea in rice.

Previous studies have reported numerous genes involved in the regulation of non-floral spikelet organ development in rice, such as *LSH1*/*OsMADS1*, *MFO1*/*OsMADS6*, *RAP1B*/*OsMADS14*, *RAP1A*/*OsMADS15*, *PAP2*/*OsMADS34*, *DP1*, *REP1*, *EG1* and *G1*. Mutation in these genes causes severe morphological alterations in various spikelet organs. For example, in the *mfo1* mutant, the identity of palea and lodicule is disturbed, and mosaic organs were observed. The number of vascular bundles increased to five to six in the *mfo1-1* palea and the interlocked lemma/palea structure was destroyed^28,49,50^. In the *lsh1* mutant, palea and lemma are elongated and leafy that exhibits a feature of open hull^22^. Detailed histological analyses demonstrate clearly that *LSH1*/*OsMADS1* regulates lemma development by controlling epidermal cell fate and proliferation and by influencing internal cell differentiation. In the palea, *LSH1*/*OsMADS1* largely regulates internal cell layer differentiation^51^. In the *pap2* mutant, elongation of sterile lemmas and rudimentary glumes was observed in all spikelets examined^52^. These genes are associated with meristem function and affect spikelet formation at the early stages by controlling cell number. In contrast, in the *rep1* mutant, the development of palea is significantly retarded and its palea exhibits vascular pattern similar to that of lemma and the mutant floret presents bilateral symmetry along the Le/Pa axis. Detailed histological analyses demonstrate clearly that loss of function of REP1 may dramatically affect the palea cell growth and expansion, especially in the epicuticula, the innermost cell layer, and vascular tissues^30^. In our study, the *th1* mutation only resulted in a slight change in the shape of the lemma and palea. SEM observation of early stages of floret development in several allelic mutants of *TH1*, such as *bls1-1 and afd1*, showed that *TH1* does not function at stages of floral-organ initiation and patterning but is required for lateral development of the lemma and palea in the final stages^34–36^. Our observation that *TH1* controls cell size during the lateral development of lemma and palea is consistent with previous finding that *TH1* is not associated with meristem function.

TH1 belongs to the ALOG family protein, which has 10 and 11 members in the rice and *Arabidopsis* genome, respectively^40^. One previous bioinformatic study has predicted that the ALOG family protein may act as transcription factors or recruiters of repressive chromatin^42^. Three ALOG family members have been functionally characterized, i.e., the *Arabidopsis* LSH1^41^, the rice G1 and TAW1 protein^32,40^. All these three proteins are shown be localized into the nucleus. The rice G1 and TAW1 are shown further to have slight but significant activity as a transcriptional activator^32,40^. In this study, we showed that TH1 is also nuclear-localized (Fig. 5) and has the ability to form the homodimer (Fig. 6), which is consistent with the attributes of most transcription factors. Homodimerization of TH1 seems to be mediated by the ALOG domain, because all three point mutations in the ALOG domain abolished its ability to form the homodimer (Fig. 6). Interestingly, we found that TH1 repressed rather than enhanced transcriptional activity in rice protoplasts using a trans-activation assay based on the yeast GAL4 system (Fig. 7). The transcriptional repression activity of TH1 was relieved by the His129Tyr substitution in the *s2-89* mutant (Fig. 7). Fusion of exogenous EAR transcription suppression domain to the mutant protein TH1^s2-89^ could largely complemented the narrow spikelet phenotype, whereas fusion with the exogenous VP16 transcription activation domain enhanced the narrow spikelet phenotype (Fig. 8). These results suggest that TH1 is involved in transcriptional regulation as repressor. However, noticeable transcription repression domains such the EAR motif^48^ could not be identified within the TH1 protein (Fig. 4). Previously, the ALOG domain was postulated to function as a DNA-binding domain derived from a novel class of DIRS-1-like retrotransposons^42^. If this is the case, binding of the ALOG proteins to target DNA might create a repressive chromatin state thus suppress transcription of the target gene. Further studies will be required to identify the target genes regulated by TH1. TH1 likely functions as an upstream modulator of cell expansion *via* the regulation of downstream target genes during rice lemma and palea development.

Some previously reported allelic mutants such as *th1-2* ^33^, *bls1-1*, *bls1-2* ^35^ have large-fragment deletion in which the *TH1* gene is completely deleted. Other allelic mutant such as *th1-1* ^33^, *th1-6569* ^34^, *bsg1-1* ^37^ and *afd1* ^36^ have small-fragment deletion which cause frame-shift mutation in the *TH1* gene. These deletion mutants provide little information about the functional domains and critical amino acids required for the proper function of the TH1 protein. In this study, the *s2-89*, *xd281* and *xd425* mutant all contain missense point mutations in the ALOG domain. The three TH1 mutant proteins TH1^s2-89^ (His129Tyr), TH1^xd281^ (Arg80Trp) and TH1^xd425^ (Gly124Gln) lost the ability to interact with itself (Fig. 6), indicating that Arg80, Gly124 and His129 are critical for the homodimerization of TH1. Moreover, we found that the His129Tyr substitution relieved the transcriptional repression activity of TH1 (Fig. 7c). Therefore, our study identified several critical amino acids in the ALOG domain essential for the TH1 function.

## Methods

### Plant materials and growth conditions

Four rice mutants, *s2-89*, *xd151*, *xd281* and *xd425*, with beak-like spikelet were identified from an M_2_ population mutagenized with ethyl methane sulfonate (EMS). The *s2-89* mutant was derived from *japonica* cv. Nipponbare and *xd151*, *xd281* and *xd425* from *japonica* cv. Xu Dao3. Corresponding cultivars were used as the wild type strains for phenotype comparison. The *s2-89* mutant was crossed with the *indica* variety Dular for genetic analysis and gene mapping. All of the parents, F_1_ hybrids and corresponding F_2_ individuals were grown in paddy fields at Beijing and Hainan under natural conditions.

### Analysis of the mutant phenotype

Plant phenotypes were photographed with a Canon digital camera. Harvested rice grains were air-dried and stored at room temperature before testing. At least 30 mature grains were measured for grain length, width and thickness using calipers. Grain weight was calculated on the basis of 200 grains and converted to 1,000-grain weight.

### Scanning electron microscopy (SEM) observation

The rice spikelet samples of the wild type and mutant were harvested at heading stage in normal growth conditions. The rice spikelet samples were fixed in 3.5% glutaraldehyde solution and then dehydrated through an ethanol series. After dehydration process, the samples were dried by critical-point drying method and sputter-coated with platinum, and then observed using a variable pressure scanning electron microscope (Hitachi, Tokyo).

### Histological analysis and GUS staining

The rice young spikelet hulls of wild type and the mutant at booting stage were fixed in FAA solution at 4 °C overnight, dehydrated in a graded ethanol series, and substituted with xylene. Finally, the samples were embedded in paraplast (Sigma, St. Louis, MO). Thin sections (8-10 μm) were prepared using a rotary microtome, dewaxed in xylene, hydrated through a graded ethanol series, stained with 1% fast green and observed using a light microscope and photographed. The cell length and width of lemma and palea were measured using the Image J software.

The histochemical GUS activities were assayed according to methods described previously^53^. Young spikelet tissues of the *CYCB1;1-GUS* transgenic rice plants were vacuum-infiltrated for 30 min in GUS staining buffer. After overnight incubation in darkness at 37 °C, the samples were completely cleared with a graded ethanol series and then photographed.

### Map-based cloning

The *s2-89* mutant was crossed with the *indica* variety Dular to develop the F_2_ population. Mutant individuals showing the beak-like spikelet in the F_2_ population were used for map-based cloning. Primary mapping was conducted with 180 pairs of InDel markers using 32 F_2_ mutant individuals. Fine mapping was performed using new markers developed based on genomic polymorphism between Dular and Nipponbare. The candidate gene was amplified and sequenced from both the *s2-89*, *xd151*, *xd281* and *xd425* mutants and wild type genomic DNA. The primer sequences for the INDEL markers and sequencing the candidate gene are listed in the Supplementary Table S3.

### RNA extraction and quantitative real-time PCR analysis

Total RNA was extracted from various rice tissues and organs of the wild type and mutant plants using TRIzol reagent (Invitrogen, Carlsbad, CA, USA) and was reverse transcribed using the M-MLV Reverse Transcriptase kit (Invitrogen), following the manufacturer’s instructions. Quantitative real-time PCR analysis was performed with a SYBR Premix Ex Taq2 (TaKaRa) kit and run on an Applied Biosystems 7500 Real-Time PCR System. The rice *Ubiquitin* gene (*LOC_Os03g13170*) was amplified as an internal control for loading normalization. The amplification program was as follows: 95 °C for 3 min, followed by 40 cycles at 95 °C for 30 s, and 60 °C for 1 min. All primers used are listed (see Supplementary Table S3). Relative transcript levels were calculated using the 2^−∆∆Ct^ quantification method^54^.

### Plant expression vector construction and rice transformation

To make the genomic DNA complementation vector, the 3,872 bp genomic DNA fragment including 2,354 bp upstream of start codon and 771 bp downstream of stop codon was amplified from the wild type Nipponbare using primers TH1-CF/R (see Supplementary Table S3) and cloned into the *EcoR*I and *Pml*I sites of the plant expression vector pCAMBIA1305.1. To make plant expression vectors harboring *EAR-TH1* ^*s2-89*^ or *VP16- TH1* ^*s2-89*^, the full-length coding sequence of *TH1* was amplified from the *s2-89* mutant using primers attB-TH1F/R (see Supplementary Table S3). PCR product was cloned into the plant expression vector LP041nEAR-hyg-asRED or LP042nVP64-hyg-asRED using the Gateway system^55^, in which four copies of the transcriptional repression motif EAR or activation motif VP16 were fused at the N-terminus of TH1. The genomic DNA complementation, *EAR-TH1* ^*s2-89*^ and *VP16- TH1* ^*s2-89*^ constructs were transformed into the *s2-89* mutant using an *Agrobacterium Tumefaciens*-mediated transformation method. The *CYCB1; 1-GUS* fusion construct was constructed as described^56,57^. A translational fusion of the 1990 bp fragment upstream of the *OsCYCB1;1* (*LOC_Os01g59120*) start codon and a 912 bp fragment of the ORF, starting at the ATG start site, which encodes an N-terminal 124 amino acids containing a mitotic degradation box, was amplified using primers CYCB1;1 F/R (see Supplementary Table S3). The 2902 bp PCR product was digested with *Hind*III and *Nco*I, and inserted into the binary vector pCAMBIA1305.1 as an in-frame fusion with the *GUS* gene. The *CYCB1; 1-GUS* fusion construct was transformed into the *s2-89* heterozygous mutant. GUS staining was conducted in the T_1_ plants showing the wild type and *s2-89* mutant phenotype, respectively.

### Transcription activation activity assay in a yeast two-hybrid system

The full-length *TH1* coding region from the wild type and the mutants was amplified by PCR using primer BD-TH1F/R (see Supplementary Table S3) and cloned into the pGBKT7 vector (Clontech). The resultant constructs were co-transformed with the empty pGADT7 vector (Clontech) into yeast strain AH109 containing the GAL4-UAS-β-galactosidase reporter gene. The transformants were grown on the solid medium lacking leucine and tryptophan. The β-Galactosidase activity was detected using the Colony-lift Filter Assay according to the manufacturer’s user manual. For dimer formation assay, the full-length *TH1* coding region from the wild type and the mutants was amplified by PCR using primer AD-TH1F/R (see Supplementary Table S3) and cloned into the pGADT7 vector. The resultant constructs were co-transformed with the pGBKT7 vector containing different *TH1* alleles into yeast strain AH109. The transformants were grown on selective solid medium SD-Leu-Trp (DDO) or SD-Leu-Trp-His-Ade (QDO). Yeast colony co-transformed with pGBKT7-53 and pGADT7-T (Clontech) was used as positive control, while yeast colony co-transformed with pGBKT7-Lam and pGADT7-T (Clontech) was used as negative control.

### Transient expression assays

Protoplast isolation and transient expression assays were performed as described previously^58^. For subcellular localization experiments, the *TH1* coding region from the wild type and mutants was amplified using primers Actin-Prom:TH1CDS:GFPF/R (see Supplementary Table S3) and ligated into the *Spe*I and *Nco*I sites of the transient expression vector pAN580, which contains the open reading frame of enhanced green fluorescent protein (EGFP) driven by the CaMV 35 S promoter^59^. The recombinant plasmids were introduced into protoplasts isolated from wild type rice seedlings and the transformed protoplasts were incubated at 28 °C for 16 h. Green fluorescence of the GFP fusion protein was observed under a Zeiss LSM 510META confocal microscope.

The transcriptional activity of TH1 was examined using the luciferase transient expression assays in rice protoplasts. The reporter plasmid Pro35S-GAL4:LUC contains a CaMV 35 S promoter, 5 × GAL4 binding site, a TATA box in front of the firefly *luciferase* (*LUC*) gene and a nopaline synthase terminator^46^. The empty effector plasmid p35S-GAL4BD:VP16 and p35S-GAL4BD:EAR (see Supplementary Figure S1) was adapted from the pSAT-GAL4DB plasmid^60^. The coding sequence of *TH1* from the wild type and *s2-89* mutant was inserted into the *Bgl*II and *BamH*I sites to generate the p35S-GAL4DB-TH1 construct, or the *Bgl*II and *Kpn*I sites to generate the p35S-GAL4DB-TH1-VP16 and p35S-GAL4DB-TH1-EAR constructs. Each transformation used 5 μg of reporter plasmid, 4 μg of effector plasmid and 1 μg of p35S: REN plasmid expressing the *Renilla* luciferase as an internal control. The activities of firefly and *Renilla* luciferases were measured sequentially from a single sample using the Dual-Luciferase Reporter assay kit (Promega, Cat. no. E1910). The relative activity of experimental reporter was expressed as the LUC/REN ratio.

### Data availability

All data generated or analysed during this study are included in this published article (and its Supplementary Information files).

## Electronic supplementary material

supplementary information
