# Supplementary material for: The rice TRIANGULAR HULL1 protein acts as a transcriptional repressor in regulating lateral development of spikelet
Source: Sci Rep. 2017 Oct 20;7:13712. doi: 10.1038/s41598-017-14146-w (PMC5651839; doi:10.1038/s41598-017-14146-w)
Supplement: Supplementary file 1 — supplementary information [file 41598_2017_14146_MOESM1_ESM.doc]

**The rice TRIANGULAR HULL1 protein acts as a transcriptional repressor in regulating lateral development of spikelet**

Peng Peng^1^, ^#^, Lihua Liu^1^, ^#^, Jingjing Fang^1^, Jinfeng Zhao^1^, Shoujiang Yuan^2^, Xueyong Li^1^, *

^1^ National Key Facility for Crop Gene Resources and Genetic Improvement, Institute of Crop Science, Chinese Academy of Agricultural Sciences, Beijing 100081, China

^2^ Shandong Rice Research Institute, Jinan 250100, China

^#^ These authors contributed equally to this work.

* Correspondence and requests for materials should be addressed to X.L. (E-mail: lixueyong@caas.cn)

**Supplementary Information**


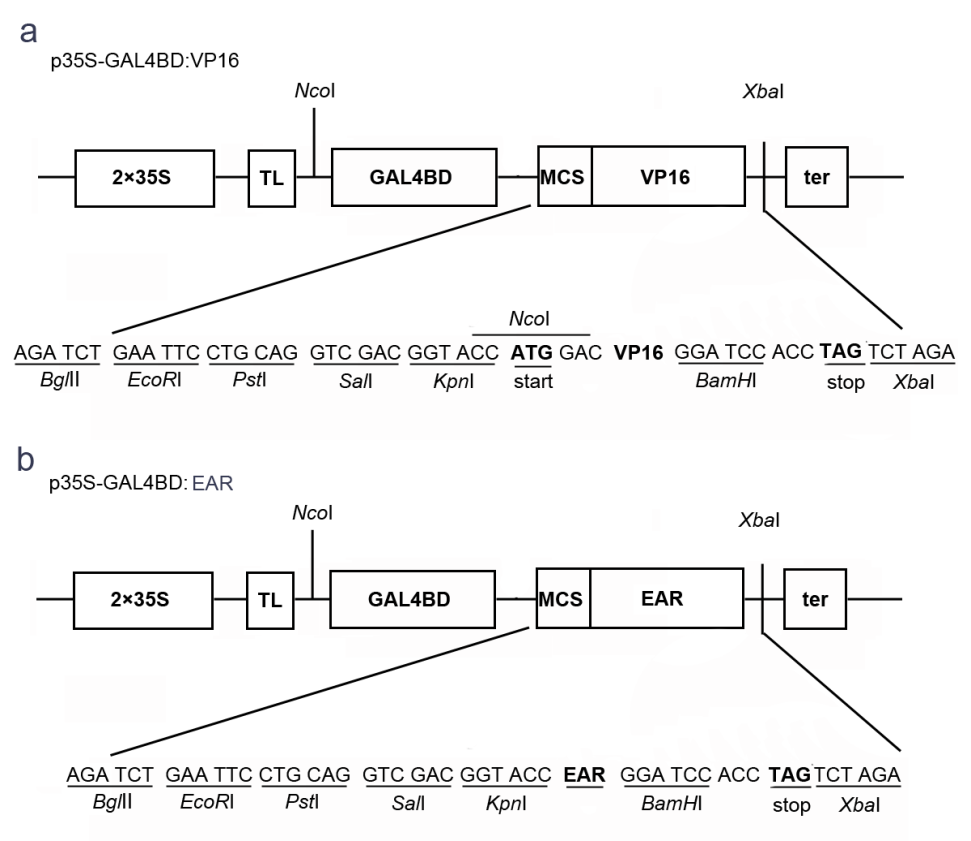


Supplementary Fig S1 Schematic presentations of the vector p35S-GAL4BD:VP16 (a) and p35S-GAL4BD: EAR (b)


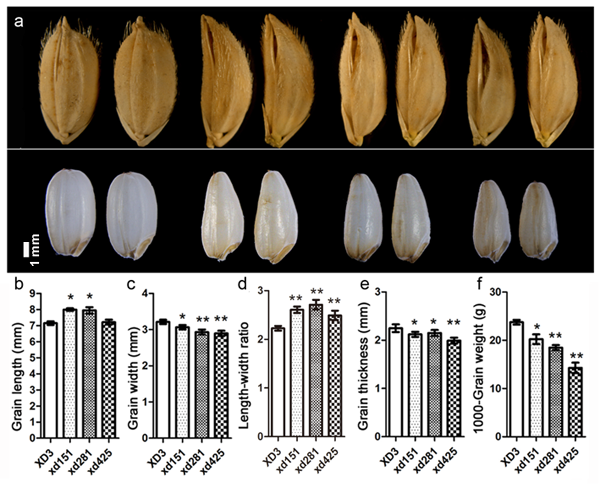


Supplementary FigS2 Grains phenotypes of the *xd151*, *xd281*and *xd425* mutants and the wild type Xu Dao3 (XD3)

(a) Grain morphology. The upper row: unhulled seeds; the lower row: hulled seeds.

(b) Comparisons of grain length, grain width, length-width ratio, grain thickness and 1000-grain weight in WT and the mutant lines.

Data are means ± SE (n=20). Double asterisk and Asterisk indicate significant differences between WT and *s2-89* at P < 0.01 and P < 0.05 by Student’s t test. Scale bar is indicated.


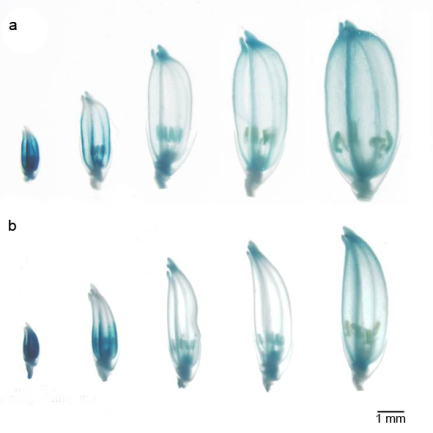


Supplementary FigS3 Histochemical staining analysis of the cell division marker reporter *OsCYCB1; 1-GUS* in the spikelet hulls at different developmental stages in the wild-type (a) and *s2-89* mutant (b) background, scale bar is indicated.


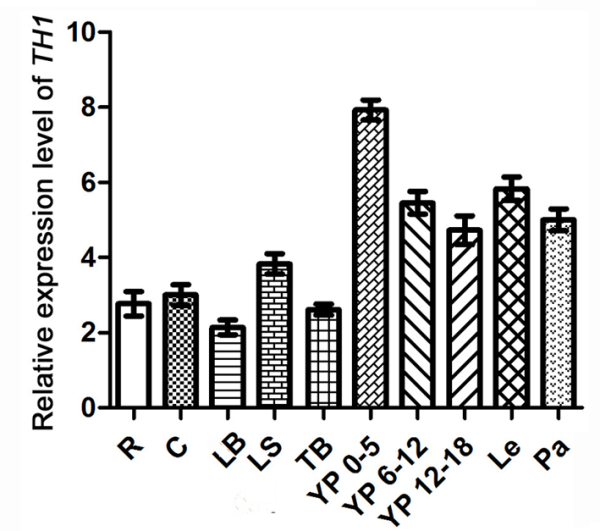


Supplementary FigS4 *TH1* expression pattern in different tissues and organs Quantitative real-time PCR analysis of the *TH1* gene expression in different tissues and organs R, root; C, culm; LB, leaf blade; LS, leaf sheath; TB, tiller bud; YP 0-5, 6-12, and 12-18 represent young panicle in length from 0-5cm, 6-12cm and 12-18cm; Le, lemma; Pa, Palea.

**Supplementary Table S1** Agronomic characters of WT (Nipponbare) and *s2-89*

| **Traits** | **WT** | ***s2-89*** | **P-Value** |
| --- | --- | --- | --- |
| Number of tillers | 17.67±2.18 | 17.84±2.82 | 0.81 |
| Panicle length (cm) | 93.11±2.95 | 89.06±3.24* | 0.018 |
| The second leaf length (cm) | 54.43±3.47 | 54.95±2.93 | 0.95 |
| The second leaf width (mm) | 13.18±0.77 | 13.17±0.59 | 0.99 |
| Panicle length (cm) | 19.99±1.11 | 20.91±0.84** | 6.60E-03 |
| No. of spikelet per panicle | 149.15±14.21 | 139.31±11.93** | 8.84E-04 |
| No. of grain per panicle | 135.70±5.92 | 119.9±5.80** | 1.12E-04 |
| Grain length (mm) | 7.42±0.078 | 7.36±0.32 | 0.35 |
| Grain width (mm) | 3.23±0.094 | 2.58±0.062** | 1.98E-23 |
| Length width ratio | 2.29±0.064 | 2.86±0.058** | 3.25E-27 |
| Grain thickness (mm) | 2.18±0.066 | 1.80±0.08** | 7.34E-18 |
| 1,000-grain weight (g) | 23.90±0.026 | 12.84±0.010** | 5.09E-09 |

Data shown are mean ± SE from 20 plants. ** indicates significant difference at P < 0.01 level,* indicates significant difference at P < 0.05 level by Student’s t test.

**Supplementary Table S2** Grain characters of *xd151*, *xd281*, *xd425* and WT (Xu dao 3)

| **Trait** | **WT** | ***xd151*** | ***xd281*** | ***xd425*** |
| --- | --- | --- | --- | --- |
| Grain Length (mm) | 7.15±0.11 | 7.99±0.083** | 7.94±0.198** | 7.21±0.15 |
| P-value |  | 6.01×10^-25^ | 4.67×10^-16^ | 0.156 |
| Grain width (mm) | 3.21±0.059 | 3.07±0.060 | 2.93±0.072** | 2.90±0.07** |
| P-value |  | 2.06×10^-9^ | 4.10×10^-16^ | 1.21×10^-17^ |
| Grain thickness (mm) | 2.25±0.081 | 2.15±0.062** | 2.12±0.053** | 1.99±0.063** |
| P-value |  | 1.01×10^-4^ | 1.11×10^-6^ | 2.09×10^-13^ |
| Length-width ratio | 2.23±0.048 | 2.61±0.065** | 2.71±0.10** | 2.49±0.096** |
| P-value |  | 2.77×10^-21^ | 8.85×10^-18^ | 1.10×10^-11^ |
| 1,000-grain weight (g) | 2.38±0.0097 | 2.02±0.013** | 1.85±0.017** | 1.43±0.014** |
| P-value |  | 4.61×10^-21^ | 1.20×10^-21^ | 8.22×10^-28^ |

Data shown are mean ± SE from 20 plants. ** indicates significant difference at P < 0.01 level,* indicates significant difference at P < 0.05 level by Student’s t test.

**Supplementary Table S3** Primers used in this study

| Primer | Forward | Reverse |
| --- | --- | --- |
| Molecular markers used for gene mapping | | |
| R2-14 | 5'-GGAAGCTTCAGCCTCACG-3' | 5'-GGTTATACAACGGCGGATCT-3' |
| R2-15 | 5'-AGTGAAATTTGAGCCCAACG-3' | 5'-TAAAAGCAAAGGCCGAAAAA-3' |
| R2-16 | 5'-CAATGGGTTCTTGGGAGATA-3' | 5'-TCACATCATTTGAGGCAAGC-3' |
| C2-1 | 5'-CCTGTCTCCGAGATGCTAAT-3' | 5'-ATCAAAATGAACAGGCCCTA-3' |
| C2-2 | 5'-TACTTGTGTAGTTGTGTGCA-3' | 5'-TCAAACCCTAGCATCCCAAT-3' |
| C2-3 | 5'-CTAGCAAACTTCAGCCTCAG-3' | 5'-CGGTACTCATAGGCATAAAT-3' |
| C2-4 | 5'-GCTGTAGACCATTGACTGGT-3' | 5'-AGATACGACATAGTTTCCCG-3' |
| C2-5 | 5'-ACGTCCGTAACCTGTCGCTA-3' | 5'-GTTGCAAGTAGCAGAGCAGT-3' |
| *TH1* gene full length DNA sequence amplification | | |
| *TH1*-SEQ1 | 5'-CATCACCAATAGTGAGACAC | 5'-CGCCGTAATGCAACCTGAAT-3' |
| *TH1*-SEQ2 | 5'-GTTCATCATCCCGTGAGTTC | 5'-CGTACTAGTGCACACCCAAT-3' |
| Primers for qPCR analysis and GUS staining | | |
| *OsUbiquitin1* | 5'-AGAAGGAGTCCACCCTCCACC-3' | 5'-GCATCCAGCACAGTAAAACACG-3' |
| *OsTH1* | 5'-ATTGGAGGATCGCTAGCTAG-3' | 5'-TGACCTTCGTCCTAGCTTAG-3' |
| *CYCB1;1* | 5'-atggtcaagcttcagttgtgtgggagccgt-3' | 5'-gctctggcccatggcagagctgatctcgatgacatg-3' |
| Primers for a yeast two-hybrid system | | |
| AD-TH1 | 5'-GGAGGCCAGTGAATTCATGAAGCGCGAGTACCAAGAAG-3' | 5'-CGAGCTCGATGGATCCTCACGCCGCGGCGACGCGCCAT-3' |
| BD-TH1 | 5'-CATGGAGGCCGAATTCATGGATCGTCACCATCACCA-3' | 5'-GCAGGTCGACGGATCCTCACGGGATGATGAACTGCG-3' |
| Primers for nuclear localization | | |
| Actin-Prom:TH1CDS:GFP | 5'-agcccagatcaACTAGTAGGatgGATCGTCACCATCAC-3' | 5'-CCCTTGCTCACCATGGATCCTAACGGGATGATGAACTGCGG-3' |
| Primers for complementation experiment | | |
| TH1-CF/R | 5'-ACATGATTACGAATTCTTGTCTCCTGCATGTATGTGC -3' | 5'-GTCACCAATTCACACGTGGCATTGCACACAGATCATAGC -3' |
| Primers for transient expression assays | | |
| GAL4DB-TH1 | 5'-TGTATCGCCGAGATCTatgGATCGTCACCATCACCACCAC-3' | 5'-TAGACTAGGTggatcctcaCGGGATGATGAACTG-3' |
| GAL4DB-  VP16-TH1 | 5'-TGTATCGCCGAGATCTatgGATCGTCACCATCACCACCAC-3' | 5'-TCCAGCGCGTCCATGGTACCCGGGATGATGAACTG-3' |
| GAL4DB-  VP16-EAR | 5'-TGTATCGCCGAGATCTatgGATCGTCACCATCACCACCAC-3' | 5'-TCCAGATCGAGCATggtaccCGGGATGATGAACTG-3' |
| attB-TH1 | 5'-GGGGACAAGTTTGTACAAAAAA GCAGGCTCAatgGATCGTCACCATCACCAC-3' | 5'-GGGGACCACTTTGTACAAGAAAGCTGGGTCgcattgcacacagatcatagc-3' |
